# Salisbury and District Infirmary and Hospital League

**Published:** 1922-09

**Authors:** L. S. Luckham

**Affiliations:** President, Infirmary League, Salisbury


### Salisbury and District Infirmary and Hospital League.

*To the Editor of* THE HOSPITAL AND HEALTH REVIEW.

SIR,—After much consideration, and after a great deal of help from our friends at Winchester, King's College Hospital, the Radcliffe, Leicester, &c., we launched our contributory scheme about 18 months ago. By contributing 1d. weekly (for young persons earning less than £1 per week), 2d. weekly (for an individual man or woman), 4d. weekly (for a man, his wife, and any children under wage-earning age) membership of the League is enjoyed, and should it be necessary for a member to become a patient of Salisbury Infirmary, *or any other hospital in the country*, the League pays such hospital 10s. 6d. on account of maintenance each week he (or she) is detained there. Out of its surplus funds—after providing for the maintenance referred to—the League hands donations to the Infirmary at intervals, such donations during the past twelve months reaching a total of £1,000.

Certain points have arisen which may be of interest to your readers, and I hope will lead to a profitable discussion. Our experience shows that contributors do expect some benefit from their subscriptions, and that any scheme, to be a success, must embody some return. We do it by paying maintenance money to any hospital or convalescent home of which the contributor may become a patient. One difficulty has to be overcome, arising from the fact that any general hospital draws patients from areas in which cottage hospitals and other general hospitals are interested. It is, of course, waste of effort for two or three appeals to be made in the same district. We have discussed this with Winchester, Southampton and some of the cottage hospitals, and hope that the result may be fruitful of benefit to all concerned. As we pointed out to the Governors of the Westminster Cottage Hospital, Shaftesbury, they are financially sound because a considerable proportion of hospital expense in their area is borne by us without an adequate contribution from that area. This, I fancy, is fairly common, but would be obviated by our co operative suggestions if worked properly.

We find that the contributory scheme is generally taken up very heartily, and that if the right people are approached there is no difficulty in getting excellent officers and collectors. Two villages, Winterslow and Whiteparish, of just over 800 in population, subscribe well over £100 a year respectively from contributory members, and in Winterslow, out of 220 houses, 180 contribute. In the city itself we have many keen members, and at our monthly Sunday meetings we not only do business but occasionally are able to get a speaker to talk of hospital matters, and so give them an insight into the management, routine and difficulties of hospitals.

Although we have no income limit, it is an understood thing that the benefits are confined to that class already recognised as suitable hospital patients.

Yours faithfully,
L. S. LUCKHAM,
President, Infirmary League, Salisbury.

